# Risk factors and clinical outcomes of severe postoperative hypoxaemia following surgical repair of Stanford type A aortic dissection: a retrospective cohort study

**DOI:** 10.3389/fmed.2025.1603262

**Published:** 2025-10-08

**Authors:** Min Lin, Yuping Xiang, Tianhui Luo, Ling Zeng, Jingxiu Fan

**Affiliations:** ^1^Department of Critical Care Medicine, West China Hospital, Sichuan University/West China School of Nursing, Sichuan University, Chengdu, Sichuan, China; ^2^Department of Cardiovascular Surgery, West China Hospital, Sichuan University, Chengdu, Sichuan, China

**Keywords:** TAAD, severe postoperative hypoxaemia, risk factors, retrospective cohort study, clinical outcomes

## Abstract

**Objective:**

This study aimed to investigate independent risk factors of severe postoperative hypoxaemia in patients with type A aortic dissection (TAAD), for providing a reference for clinical healthcare professionals to identify high-risk patients early and formulate postoperative severe hypoxaemia prevention and intervention plans.

**Methods:**

This was a retrospective, observational study, patients with Stanford TAAD who underwent aortic surgery (*n* = 755) between March 2021 and July 2024 were enrolled. Relevant preoperative and intraoperative clinical data were collected. Univariate and logistic regression analyses were used to identify risk factors of severe hypoxaemia after TAAD.

**Results:**

Of the 755 patients with postoperative TAAD, 192 (25.43%) experienced severe postoperative hypoxaemia. Multivariable logistic regression analysis revealed body mass index (BMI) (odds ratio [OR] = 1.137), white blood cell count (WBC) (OR = 1.068), hypertension (OR = 2.693), chronic kidney disease (CKD) (OR = 2.767), highest lactate level (OR = 1.094) as independent predictors of severe postoperative hypoxaemia. Femoral artery cannulation (OR = 0.533) was a protective factor against severe postoperative hypoxaemia. Severe hypoxaemia increased hospitalization costs, prolonged mechanical ventilation duration, and extended intensive care unit and hospital stay durations.

**Conclusion:**

The high severe hypoxaemia prevalence after TAAD is associated with poor clinical outcomes. Preoperative BMI, WBC, hypertension, CKD and intraoperative highest lactate level were risk factors for severe postoperative hypoxaemia. Clinical healthcare professionals should closely monitor high-risk patients, intensify oxygenation index surveillance, and intervene promptly to minimize hypoxaemia and improve patient prognosis.

## Introduction

Stanford type A aortic dissection (TAAD), which is defined as a dissection involving the ascending aorta with possible extension to the aortic arch or brachiocephalic artery, is a life-threatening condition requiring immediate open surgical intervention owing to its association with severe morbidity and mortality (1). Recent studies have shown that the incidence of TAAD is approximately 2.6 to 7.2 per 100,000 person-years (2, 3). Without surgical intervention, mortality rates reach 50% within the first 48 h; hence, guidelines recommend urgent surgery in all patients (4–6); data from the International Registry of Aortic Dissection (IRAD) indicates that an overall in-hospital mortality rate of 16.7–31.4% (7). Survival rates following TAAD have significantly increased in the past 20 years owing to improved surgical, technical, and perioperative management; however, the incidence of postoperative complications remains high (6, 8). Postoperative hypoxaemia, characterized by respiratory failure, remains a common TAAD complication after surgery, sample sizes range from 60 to 403, with an incidence ranging from 16.7 to 68.75% (9). Postoperative hypoxaemia is followed by prolonged mechanical ventilation, increased length of stay in the intensive care unit (ICU) and hospital, increased hospitalization costs, damage to the lung and other organ functions, and even increased mortality (10–12). Therefore, preventing postoperative hypoxaemia after TAAD is essential. Several studies have focused on the risk factors for postoperative hypoxaemia (10, 11, 13); however, few have focused on the risk factors for severe hypoxaemia (PaO_2_/FiO_2_ ratio < 100 mmHg) after TAAD (14–17); sample sizes ranged from 162 to 492, moreover, the risk factors included were not comprehensive. Our center is the largest aortic dissection surgery center in Southwest China, the cases were well represented, in this study, to investigate risk factors for severe postoperative hypoxaemia (defined as PaO2/FiO2 < 100 mmHg) and to provide guidance for preventing this complication. In addition, the variables were screened based on the results of a meta-analysis conducted by our team and expert consultation, in order to identify high-risk patients early and accurately, our study only included preoperative and intraoperative possible 54 variables. These objectives logically lead into a retrospective study design that would involve collecting relevant patient data, performing statistical analyses (univariate and logistic regression), and identifying independent risk factors.

## Methods

### Study design and settings

This single-center retrospective observational clinical study enrolled consecutive patients who underwent TAAD repair at the our center between March 2021 and July 2024. The study was conducted in accordance with the Declaration of Helsinki and was approved by the Ethics Committee (Number ID: 2024 Annual Audit 848). The requirement for written informed consent was waived because of the observational and retrospective nature of this study.

### Study population

The study comprised 755 consecutive adult patients (age ≥ 18 years) (573 [75.89%] males) who underwent TAAD repair and were hospitalized during this period in the ICU after surgery. Patients who died intraoperatively and those with missing data were excluded. The diagnostic criteria for hypoxaemia according to the Berlin Criteria for acute respiratory distress syndrome (18) were mild (200 mm Hg < PaO_2_/FiO_2_ ≤ 300 mm Hg), moderate (100 mm Hg < PaO_2_/FiO_2_ ≤ 200 mm Hg), and severe (PaO_2_/FiO_2_ ≤ 100 mm Hg) hypoxaemia. In the present study, postoperative severe hypoxaemia was defined as PaO_2_/FiO_2_ ≤ 100 mmHg. Collected data on hypoxaemia in the first 72 h after surgery, blood gas analyses 4–6 times daily in patients after TAAD surgery, calculating the lowest oxygen index in 72 h. The 755 patients were divided into two groups, namely severe hypoxaemia (*n* = 192) and non-hypoxaemia (*n* = 563) groups.

### Data collection

Clinical data were collected using the electronic medical record management system of the hospital. Preoperative variables in this study were as follows: demographic variables such as age, gender, body mass index (BMI), smoking history and drinking history; underlying conditions such as hypertension, diabetes mellitus, coronary heart disease, stroke, chronic kidney disease (CKD), chronic lung disease, combined valve diseases, and history of cardiovascular surgery; radiological examination, including primary tear and deBakey type of aortic dissection; laboratory tests Pre-operative Collection of the results of the most recent tests conducted before operation, including hemoglobin, white blood cell count, neutrophil percentages, d-dimer, bilirubin, high-density lipoprotein, low-density lipoprotein, triglyceride, uric acid, serum creatinine, blood glucose, albumin, alanine transaminase, aspartate transaminase, and myoglobin.

Intraoperative variables were as follows: emergency surgery, aortic arch replacement, combined valve surgery, coronary artery bypass grafting, descending aortic stent implantation, cannulation site (which is used to establish cardiopulmonary bypass, for example, axillary artery, femoral artery, direct aorta artery), cerebral perfusion, operation time, cardiopulmonary bypass (CPB) time, aortic cross-clamping time, hypothermia temperature, blood transfusion volume, highest glucose levels, highest lactic acid levels, and deep hypothermic circulatory arrest time. Highest lactic acid defined as the highest lactate value measured by blood gas analysis after the start of CPB.

Postoperative variables were the total costs, duration of mechanical ventilation, length of ICU stay, length of hospitalization, and self-discharge or mortality. Mortality is defined as postoperative all-cause deaths that occurred in the hospital.

### Statistical analysis

Statistical analyses were performed using SPSS version 22.0. The Kolmogorov–Smirnov test was used to evaluate whether continuous variables were normally distributed. Continuous variables were expressed as mean ± standard deviation when normally distributed and as median (25th–75th percentiles) when skewed. Categorical variables are presented as absolute values and relative frequencies. Univariate logistic regression analysis was conducted to screen for potential risk factors of severe postoperative hypoxaemia. Normally distributed continuous variables with homogeneous variance were compared using Student’s t-test; otherwise, the Mann–Whitney U test was used. Categorical variables were compared using chi-squared or Fisher’s exact test. Similarly, a multivariate logistic regression analysis model (forward stepwise) was employed to test for independent predictors of severe hypoxaemia for variables with statistical significance in the univariate analysis (*p* < 0.05), and the odds ratio (OR) was calculated with a 95% confidence interval (CI). The clinical outcomes were compared between the severe hypoxaemia and non-hypoxaemia groups. *p*-value <0.05 was considered statistically significant.

## Results

### General clinical data

In this study, 759 patients underwent TAAD repair with cardiopulmonary bypass at the West China Hospital of Sichuan University between March 2021 and July 2024. Of these, four were excluded; one was <18 years and three died within 24 h after surgery. Therefore, 755 patients were included, with 192 (25.43%) having severe postoperative hypoxaemia.

### Preoperative data

Preoperative patient variables are presented in Table 1. These include demographic characteristics such as sex (573 males; 75.89%) and median age (51 years; interquartile range 43–60); underlying conditions such as hypertension (582; 77.09%), chronic lung disease (35; 4.64%), CKD (50; 6.62%), and history of cardiovascular disease (134; 17.75%); and radiological examination such as the location of primary tear in the ascending aorta (553; 73.24%).

**Table 1:** Preoperative variables of patients in different groups.

Variables	Total (*n* = 755)	Non-hypoxemia (*n* = 563)	Severe hypoxemia (*n* = 192)	*p* value
Demographics				
Age (years)	51 (43–60)	52 (43–60)	51 (44–59)	0.655
Males	573 (75.89)	436 (77.44)	137 (71.35)	0.089
BMI (kg/m^2^)	24.91 (22.49–27.76)	24.44 (22.04–27.06)	26.57 (24.06–29.30)	0.000*
Smoking history	295 (39.07)	227 (40.32)	68 (35.42)	0.229
Drinking history	229 (30.33)	177 (31.44)	52 (27.08)	0.257
Underlying conditions				
Hypertension	582 (77.09)	409 (72.65)	173 (90.10)	0.000*
Diabetes	24 (3.18)	15 (2.66)	9 (4.69)	0.168
Marfan syndrome	21 (2.78)	20 (3.55)	1 (0.52)	0.027*
CAD	14 (1.85)	9 (1.60)	5 (2.60)	0.372
History of stroke	25 (3.31)	19 (3.37)	6 (3.13)	0.867
Chronic lung disease	35 (4.64)	27 (4.80)	8 (4.17)	0.72
CKD	50 (6.62)	30 (5.33)	20 (10.42)	0.014*
Acute aortic coarctation	636 (84.24)	462 (82.06)	174 (90.63)	0.005*
Combined valve diseases	67 (8.87)	57 (10.12)	10 (5.21)	0.039*
Cardiovascular surgery history	134 (17.75)	105 (18.65)	29 (15.10)	0.267
Radiological examination				
Primary tear				0.006*
Ascending aorta	553 (73.24)	416 (73.89)	117 (60.94)	
Aortic arch	177 (23.44)	115 (20.43)	62 (32.29)	
Descending aorta	29 (3.84)	21 (3.73)	8 (4.17)	
Debakey (extensive type)	669 (88.61)	490 (87.03)	179 (93.23)	0.02*
Bovine aortic arch	11 (1.46)	7 (1.24)	4 (2.08)	0.484
Laboratory values				
Hemoglobin (g/L)	126.00 (113.00–139.00)	126.50 (113–138.00)	126 (109–139)	0.308
WBC (×10^9^/L)	10.55 (8.30–13.27)	10.11 (7.88–12.80)	11.55 (9.10–14.17)	<0.001*
Blood platelets	153.00 (113.00–198.50)	154.00 (113.00–200.00)	152.50 (108.00–194.00)	0.606
Neutrophil percentages (%)	81.10 (75.00–86.30)	80.30 (74.10–85.60)	83.75 (78.00–87.30)	0.006*
Absolute neutrophil values	8.58 (6.40–11.19)	8.15 (5.78–10.60)	9.58 (7.22–12.05)	<0.001*
Mononuclear cell	0.79 (0.56–1.11)	0.77 (0.55–1.08)	0.86 (0.63–1.18)	0.003*
D-dimer	5.57 (2.3–12.42)	0.25 (2.13–11.99)	7.23 (2.95–13.74)	0.009*
Bilirubin	15.20 (10.65–21.50)	15.00 (10.30–20.70)	16.15 (11.5–23.00)	0.012*
HDL (mmol/L)	1.12 (0.91–1.36)	1.13 (0.92–1.35)	1.10 (0.87–1.38)	0.341
LDL (mmol/L)	2.31 (1.78–2.84)	2.31 (1.74–2.81)	2.31 (1.89–2.84)	0.49
Triglyceride	1.16 (0.85–1.63)	1.12 (0.81–1.53)	1.27 (0.96–1.95)	<0.001*
Cholesterol (mmol/L)	3.86 (3.32–4.45)	3.85 (3.27–4.41)	3.91 (3.37–4.67)	0.442
Uric acid (μmol/L)	362.50 (284.00–447.00)	357.00 (279.00–435.00)	390.50 (303.00–471.00)	0.031*
Scr (g/L)	72.00 (72.00–116.00)	88.00 (72.00–111.00)	97 (74–133)	0.008*
Blood glucose (mmol/L)	6.75 (5.64–7.87)	6.62 (5.49–7.71)	7.17 (6.14–8.20)	<0.001*
Albumin (g/L)	39.10 (36.10–41.70)	39.10 (36.20–41.70)	38.85 (35.00–41.70)	0.337
ALT	20.00 (13.00–36.00)	19.00 (13.00–35.00)	25.00 (15.00–41.00)	0.007*
AST	20.00 (15.00–34.00)	19.50 (15.00–32.00)	23.50 (17.00–42.00)	0.03*
Myoglobin	38.20 (21.00–87.55)	35.29 (21.00–82.88)	42.00 (21.69–118.00)	0.076

Continuous variables were expressed as mean and standard deviation for normal data, and as median and interquartile range for non-normal data. Categorical variables were expressed as number and percentage. BMI, body mass index; WBC, white blood cell; CAD, coronary artery disease; CKD, chronic kidney disease; HDL, high density lipoprotein; LDL, Low-density lipoprotein; Scr, serum creatinine; ALT, alanine transaminase; AST, Aspartate transaminase. *The statistical significance was set at a *p*-value less than 0.05.

### Intraoperative data

The operative variables of the patients are listed in Table 2. There were 555 emergency surgeries (73.51%), 514 total arch replacements (68.08%), with a mean operative time of 6.8 h, extracorporeal circulation time of 187 min, highest intraoperative blood glucose level of 10 mmol/L, and highest intraoperative lactate level of 5 mmol/L.

**Table 2:** Operative variables of patients in different groups.

Variables	Total (*n* = 755)	Non-hypoxemia (*n* = 563)	Severe hypoxemia (*n* = 192)	*P*-value
Emergency operation	555 (73.51)	414 (73.53)	141 (73.43)	0.979
Aortic arch replacement				0.004*
Hemiarch	161 (21.32)	128 (22.74)	33 (17.19)	
Total arch	514 (68.08)	366 (65.01)	148 (77.08)	
Combined valve surgery				0.004*
Valvuloplasty	381 (50.46)	264 (46.89)	117 (60.94)	
Valve replacement	296 (39.21)	243 (43.16)	53 (27.60)	
CABG	64 (8.48)	48 (8.53)	16 (8.33)	0.934
Descending aortic stent implantation	529 (70.07)	375 (66.61)	154 (80.21)	0.000*
Cannulation site				0.014*
Axillary artery	124 (16.42)	84 (14.92)	40 (20.83)	
Femoral artery	587 (77.75)	447 (79.40)	140 (72.92)	
Direct aorta artery	17 (2.25)	16 (2.84)	1 (0.52)	
Two or more cannulation	27 (3.58)	16 (2.84)	11 (5.73)	
Operation time (hour)	6.80 (6.08–7.85)	6.70 (6.00–7.80)	7.00 (6.25–7.93)	0.001*
CPB time (min)	187.00 (157.00–221.00)	184.00 (156.00–221.00)	190.00 (162.00–220.00)	0.219
Aortic cross-clamping (min)	136.00 (109.00–171.00)	136.50 (108.00–172.00)	134.00 (111.00–166.00)	0.835
Hypothermia temperature (°C)	24.00 (23.40–24.70)	24.05 (23.40–24.80)	24.00 (23.50–24.70)	0.406
Red blood cell transfusion (U)	0.00 (0.00–1.50)	0.00 (0.00–1.50)	0.00 (0.00–2.00)	0.284
Plasma transfusion (ml)	0.00 (0.00–0.00)	0.00 (0.00–0.00)	0.00 (0.00–0.00)	0.716
Platelet transfusion (u)	10.00 (1.00–2.00)	1.00 (1.00–2.00)	2.00 (1.00–2.00)	0.101
Input (ml)	1,500 (1200–2000)	1,500 (1150–2000)	1,600 (1200–2,100)	0.009*
Highest glucose (mmol/L)	10.00 (8.40–12.05)	9.70 (8.20–11.70)	10.80 (8.60–12.90)	<0.001*
Highest lactate (mmol/L)	5.00 (3.60–7.50)	4.80 (3.50–7.10)	6.15 (4.20–9.70)	<0.001*
DHCA (min)	23.00 (16.00–29.00)	23.00 (15.00–29.00)	23.00 (17.00–29.00)	0.063

For categorical variables, n (%) is presented. For continuous variables, median (25th-75th percentiles) is presented. CPB, cardiopulmonary bypass; CABG, coronary artery bypass grafting; DHCA, deep hypothermic circulatory arrest.

### Risk factors for postoperative hypoxaemia

Preoperative and intraoperative variables were included in the logistic regression analysis to evaluate risk factors for postoperative hypoxaemia (Table 3). BMI (OR 1.137; 95% CI [1.081–1.197]; *p* < 0.001), WBC (OR 1.068; 95% CI [1.018–1.120]; *p* = 0.007), hypertension (OR 2.693; 95% CI [1.556–4.663]; *p* < 0.001), CKD (OR 2.767; 95% CI [1.334–5.739]; *p* = 0.006), highest lactate level (OR 1.094; 95% CI [1.043–1.147]; *p* < 0.001) were independent risk factors for severe hypoxaemia. However, femoral cannulation (OR 0.533; 95% CI [0.326–0.871]; *p* = 0.012) was a protective factor against severe postoperative hypoxaemia.

**Table 3:** Multivariable analysis of risk factors for postoperative severe hypoxemia.

Variables	Standard error	Odds ratio	95% CI	*P-*value
BMI	0.026	1.137	1.081–1.197	<0.001
WBC	0.024	1.068	1.018–1.120	0.007
Hypertension	0.28	2.693	1.556–4.663	<0.001
CKD	0.372	2.767	1.334–5.739	0.006
Highest lactate	0.024	1.094	1.043–1.147	<0.001
Femoral artery cannulation	0.25	0.533	0.326–0.871	0.012

BMI, body mass index; WBC: white blood cell; CKD, chronic kidney disease.

### Clinical outcomes

The effect of severe postoperative hypoxaemia on clinical outcomes in TAAD was assessed and analyzed. The results suggest that the proportion of self-discharge or mortality (*p* < 0.0001) was significantly higher in the hypoxaemia group than in the non-hypoxaemia group. Total costs (21.85 vs. 19.93 ten thousand yuan; *p* < 0.001), ventilation time (47 vs. 19 h; *p* < 0.001), length of ICU stay (111.80 h vs. 80.25 h; *p* <0.001), and length of hospitalization (14.00 vs. 12.00 day; *p* = 0.003) of patients in the hypoxaemia group was significantly higher than those of patients in the non-hypoxaemia group (Table 4).

**Table 4:** Postoperative complications in different groups.

Complications	Total (*n* = 755)	Non-hypoxemia (*n* = 563)	Severe hypoxemia (*n* = 192)	*P*-value
Total costs (ten thousand yuan)	20.66 (17.89–25.88)	19.93 (17.56–25.52)	21.85 (18.94–26.47)	<0.001
Duration of MV, hour	21.85 (13.86–59.55)	19.00 (13.00–44.50)	47.00 (17.70–80.30)	<0.001
ICU stay, hour	85.20 (58.85–134.10)	80.25 (54.50–114.00)	111.80 (71.30–178.00)	<0.001
Hospital stay, day	13.00 (10.00–16.00)	12.00 (10.00–16.00)	14.00 (11.00–16.00)	0.003
Outcome				<0.000
Self discharge	54 (7.15)	33 (5.86)	21 (10.94)	
In-hospital all-cause mortality	16 (2.12)	6 (1.07)	10 (5.21)	
Recover	685 (90.73)	524 (93.07)	161 (83.85)	

For categorical variables, *n* (%) is presented. For continuous variables, median (25th–75th percentiles) is presented. MV, mechanical ventilation.

## Discussion

Hypoxaemia is a common postoperative complication in patients with TAAD. The incidence of severe hypoxaemia was 25.43% (192 out of 755) in our study, which is consistent with the results of a recent study (24.2%) (15); however, it is less than those reported in the study of Guan et al. (13). Previously reported risk factors for hypoxaemia after TAAD surgery include age, smoking history, renal insufficiency, higher BMI, white blood cell count, hematocrit, PaO_2_/FiO_2_ ratio, Stanford type, pH, cardiopulmonary bypass time, postoperative lactic acid level, postoperative creatinine level, and intraoperative aortic occlusion time (11, 12, 15, 19). Our study further confirmed that preoperative BMI, WBC, hypertension, CKD, and intraoperative maximum lactate level were risk factors for severe postoperative hypoxaemia.

This study reveals that higher BMI is associated with the development of postoperative and severe hypoxaemia, which is consistent with the results of previous studies (12, 13, 15, 20). In the current study, the average BMI of the included patients was 24.91, which was consistent with previous studies. Gong et al. (21) reported an evident decrease in lung compliance and respiratory resistance in patients with obesity. In addition, the inflammatory response and oxidative stress may be involved in the process of lung injury in aortic dissection caused by obesity (22). Therefore, this study highlights the significance of preventing severe postoperative hypoxaemia in patients with high BMI. Similarly, the WBC, a biomarker that reflects the systemic inflammatory response, representing higher inflammatory responses which may contribute to respiratory dysfunction, was another independent predictor of severe hypoxaemia, it is consistent with the results of previous studies (12, 23). Additionally, 90.11% of patients in the severe hypoxaemia group had a history of hypertension, which was higher than that reported in the study of Sheng et al. (15) (73.1%). Patients need to maintain low blood pressure with post-TAAD. Guo et al.’s (24) study revealed that altered pulmonary circulation and insufficient tissue perfusion related to low blood pressure were responsible for the development of hypoxaemia.

CKD is an additional independent predictor of severe hypoxaemia in our study, which has been reported previously (10, 11, 15, 25). Zhou et al. (10) reported that the APACHE II score was independently associated with the development of severe postoperative hypoxaemia, in which renal function may play a vital role. Sheng et al. (15) reported that the inflammatory response, regulation of erythropoietin production by the kidney, and the resultant oxygen delivery may play an essential role (26).

Highest intraoperative lactate levels and cerebral perfusion were independent risk factors for hypoxaemia, the mean maximum intraoperative lactate level was 6.15 mmol/L in the hypoxaemia group. Lactic acid is an intermediate product of anaerobic glycolysis, which is metabolized in the liver and kidney primarily through glycogenosis or direct oxidative decomposition. In addition, lactic acid is primarily produced under anaerobic conditions, and tissue hypoxia can elevate its levels in the body, is a marker of tissue hypoxia. Anesthesia, cardiopulmonary bypass, and surgical procedures during TAAD can cause tissue hypoxia and increase lactic acid levels (27, 28), patients who have undergone heart surgery usually exhibit elevated lactate levels (29). Wang et al. (19) showed that median lactic acid was 3.6 mmol/L before surgery and 5.0 mmol/L after surgery in patients with acute TAAD. Maintaining sufficient oxygen delivery is the critical determinant to satisfy metabolic needs during CPB, during CPB, maintain adequate tissue perfusion tissue, increasing the pump flow, increasing hemoglobin level and decreasing temperature. Furthermore, it’s worth noting that that patients with femoral artery cannulation had a lower incidence of severe hypoxaemia. Generally speaking, femoral artery cannulation is always the first choice, less time compared with axillary artery cannulation, may quicker establishment of CPB in patients with TAAD surgery (30, 31), Wang et al. (14) showed that CPB ≥ 257.5 min was the risk of postoperative severe acute lung injury after ATAAD. In our research, the femoral artery is the most common site of cannulation (77.75%), CPB time was 187 min.

In this study, patients who experienced severe postoperative hypoxaemia presented with higher rates of self-discharge or mortality and had a longer duration of mechanical ventilation support, length of ICU stay, length of hospitalization, and higher total costs. However, consistent with previous studies (10–12), we did not follow deaths after discharge. Therefore, clinicians should intervene in the early postoperative hypoxemia according to the risk factors.

### Limitations

This study had a few limitations. First, this is a single-center retrospective study, making it subject to inherent selection and information biases. Second, preoperative and intraoperative data were collected as much as possible; however, emergency surgery accounted for 73.51% of cases, and preoperative variables, such as PaO_2_/FiO_2_ and C-reactive protein data, were missed more than 20%, therefore, inclusion in the analysis was not considered. Third, we only collected data on hypoxaemia in the first 72 h after surgery, and changes in PaO_2_/FiO_2_ ratio over time were not analyzed. Finally, outcome measures included only short-term outcomes; long-term outcomes were not followed up.

In conclusion, hypoxaemia is a common postoperative complication of Stanford TAAD. The study indicated that preoperative BMI, hypertension, CKD, white blood cell count and intraoperative maximum lactate level were independent risk factors for severe postoperative hypoxaemia in patients with TAAD. Severe hypoxaemia is closely associated with poor clinical outcomes. These results indicate that early detection of postoperative hypoxaemia may improve prognosis.

## Data Availability

The raw data supporting the conclusions of this article will be made available by the authors, without undue reservation.
